# Introducing Explorer of Taxon Concepts with a case study on spider measurement matrix building

**DOI:** 10.1186/s12859-016-1352-7

**Published:** 2016-11-17

**Authors:** Hong Cui, Dongfang Xu, Steven S. Chong, Martin Ramirez, Thomas Rodenhausen, James A. Macklin, Bertram Ludäscher, Robert A. Morris, Eduardo M. Soto, Nicolás Mongiardino Koch

**Affiliations:** 1 University of Arizona, Tucson, AZ USA; 2 Museo Argentino de Ciencias, Naturales, CONICET, Buenos Aires, Argentina; 3 Agriculture and Agri-Food Canada, Ottawa, Canada; 4 University of Illinois at Urbana-Champaign, Champaign, USA; 5 University of Massachusetts at Boston and Harvard University Herbaria, Massachusetts, USA; 6 Department of Geology & Geophysics, Yale University, New Haven, Connecticut USA

**Keywords:** Information extraction, Text mining, Natural language processing, Taxonomic morphological descriptions, Phenotypic characters, Phenotypic traits, Evaluation, Spiders, ETC, Explorer of Taxon Concepts

## Abstract

**Background:**

Taxonomic descriptions are traditionally composed in natural language and published in a format that cannot be directly used by computers. The Exploring Taxon Concepts (ETC) project has been developing a set of web-based software tools that convert morphological descriptions published in telegraphic style to character data that can be reused and repurposed. This paper introduces the first semi-automated pipeline, to our knowledge, that converts morphological descriptions into taxon-character matrices to support systematics and evolutionary biology research. We then demonstrate and evaluate the use of the ETC Input Creation - Text Capture - Matrix Generation pipeline to generate body part measurement matrices from a set of 188 spider morphological descriptions and report the findings.

**Results:**

From the given set of spider taxonomic publications, two versions of input (original and normalized) were generated and used by the ETC Text Capture and ETC Matrix Generation tools. The tools produced two corresponding spider body part measurement matrices, and the matrix from the normalized input was found to be much more similar to a gold standard matrix hand-curated by the scientist co-authors. Special conventions utilized in the original descriptions (e.g., the omission of measurement units) were attributed to the lower performance of using the original input. The results show that simple normalization of the description text greatly increased the quality of the machine-generated matrix and reduced edit effort. The machine-generated matrix also helped identify issues in the gold standard matrix.

**Conclusions:**

ETC Text Capture and ETC Matrix Generation are low-barrier and effective tools for extracting measurement values from spider taxonomic descriptions and are more effective when the descriptions are self-contained. Special conventions that make the description text less self-contained challenge automated extraction of data from biodiversity descriptions and hinder the automated reuse of the published knowledge. The tools will be updated to support new requirements revealed in this case study.

**Electronic supplementary material:**

The online version of this article (doi:10.1186/s12859-016-1352-7) contains supplementary material, which is available to authorized users.

## Background

Biologists rely heavily on a variety of publications (journals, monographs, faunas/floras, etc.) to discover prior knowledge about organisms of interest. Scientific names are the primary identifiers for organisms used within these information resources. Due to different taxonomic perspectives of authors through time, scientific names and their associated descriptions in these works are not static but represent taxonomic concepts [1]. This continuous change in taxonomic concepts thus brings into question the validity of using scientific names alone as a basis of comparison. However, taxonomic works often contain detailed morphological, distributional, and other evidence that can assist with analyzing the evolution in taxonomic concepts over time.

This morphological evidence can be managed in taxon-character matrices, a research tool widely used in biological research, ranging from taxonomy to phylogenetic studies. Traditionally, these matrices are created manually by biologists within their taxonomic area of expertise. It is a tedious and laborious process because the matrix author(s) must manually select relevant character information from published literature and/or other sources and populate the matrix with their associated character states or values. By far the most common method of making taxon-character matrices is using a spreadsheet, although other software tools, for example MacClade [2] and Mesquite [3], have been also used to make the matrix creation process more efficient. More recently MorphoBank has made a web-based matrix editor available for researchers to collaboratively develop large matrices [4].

The challenge of efficiently extracting character information from systematics publications into a structured format, such as taxon-character matrices, remains open. The Phenoscape knowledgebase and a number of model organism databases employ human curators to convert natural language phenotype character descriptions into a machine-readable form by using Phenex [5] or other web-based platforms [6–9]. These manual approaches effectively capture high quality character data. However, they are time consuming and expensive.

Automated extraction of factual information from text remains an active research area after decades of research. It was previously called message understanding in the eighties, but is now better known as information extraction, and sometimes also semantic role labelling, semantic parsing, or more generally text mining. Algorithms and software have been developed for general domains (e.g., news articles, Wikipedia articles, e-commerce), for specific domains (e.g., biomedical, engineering, patents), for different extraction targets (e.g. sentiment/emotion extraction), and for text in different human languages. Currently, the dominant overall approach is using various machine learning methods (supervised and/or unsupervised) with text syntactic analyses, various knowledge resources (e.g. frame templates, glossaries, ontologies), and when available, large corpus of unlabelled data, as well [10–12].

Portability, the ability of a natural language processing system to perform equally well in a domain different from the one for which it was trained/developed, remains the greatest challenge [10]. In-domain vs. out-domain performance differences were consistently found in all systems participated in the Semantic Role Labelling shared tasks of CoNLL 2005 and 2009 [10, 13]. This is because texts in different domains contain different features that computers need to learn. This explains the need to develop various information extraction systems for different domains and different tasks, see for example, the shared tasks offered by CoNLL for general domain (http://ifarm.nl/signll/conll/), BioCreative for biomedical domains [14–17], and BioNLP Shared Tasks (http://2016.bionlp-st.org/) for biodiversity domains.

Extracting morphological characters from taxonomic descriptions received relatively little attention, but it has made significant progress in the past decades. Taylor [18] used grammar rules and a lexicon to extract plant characters from several floras. The performance was not scientifically evaluated but estimated at 60–80% recall. Taylor noted also that different parsers (grammar + lexicons) may be needed to parse characters for other taxon groups, suggesting variations within biodiversity. Diedrich, Foruner, & Milton’s Terminator [19] was an interactive system that used fuzzy keyword matching, a set of heuristic rules, and a handcrafted dictionary of structures, characters and character states. The system was evaluated with one random description, which showed that 55% of the time a perfect structure-character-state triple was among the first five candidates extracted by the system. This work suggests that morphological descriptions are not as structured as many had expected. Wood et al., [20] sampled 42 descriptions from five plant species and their genera described in six different floras in English. They lumped all non-numerical characters, such as color and shape into one Plant Feature character. Evaluated on 18 descriptions, the system showed 66/74% recall/precision in character extraction. The first system that used a machine learning method to process plant descriptions was Cui [21] but she only parsed the text at the sentence level. Tang and Heidorn [22] subsequently advanced the research to the character level. They adapted Soderland’s supervised learning system, WHISK [23], to extract leaf characters and fruit/nut shapes from 1600 Flora of North America (FNA) species descriptions. The system scored 33–80% in recall and 75–100% in precision depending on the characters. The lower recall indicates the training examples used may not have covered the characters in the test descriptions well, suggesting a weakness in the supervised learning approach for this task. Cui, Boufford, & Selden [24] showed that an unsupervised learning algorithm was able to learn 50–93% of the structure and character state terms directly from text morphological descriptions without any training examples. At the same time, Cui [25] showed that linguistic resources – machine-readable lexicons, glossaries, or ontologies – for extracting biodiversity characters were lacking. She examined three linguistic resources for plant domain and one for biology overall, and found that they lacked coverage of domain terms, an issue that still exists today: the Phenotype Quality Ontology [26] currently contains 2300+ terms, roughly the same size as FNA Categorical Glossary [27]. In contrast, Huang et al. [28] used CharaParser [29] and extracted over 7000 unique phenotype terms from 30 volumes of FNA and Flora of China alone. These resources also lack agreement in their term categorizations (e.g. is *apex* a structure or a feature? Is *erect* an orientation or position?). The four glossaries/ontologies agreed only 19% of the time on a set of 64 core character terms extracted from two plant description resources. Research further shows there is a high likelihood of an unlimited number of character terms needed to describe the entire scope of the biodiversity domains [30]. This suggests a supervised learning approach may not be the best choice for extracting morphological characters [25]. At the same time, learning and growing consensus-based linguistic/knowledge resources for phenotype characters of biodiversity are relatively urgent tasks.

CharaParser [29] was developed to address these issues. It uses an unsupervised learning algorithm [24] to learn domain terms and the mature general-purpose parser, Stanford Parser [31], to analyze sentence structures. The learned domain terms inform Stanford Parser what the Part of Speech tags for the domain terms are to help it adapt to the domain of morphological descriptions. Evaluated on FNA and invertebrate treatises, the system performed at 85 to over 90% precision and recall on character extraction, when provided with a comprehensive glossary.

CharaParser semi-automatically extracts character information from taxonomic descriptions of various taxon groups and in the process involves biologists in categorizing domain terms (e.g. *leg* is a structure, *erect* is orientation). The categorized terms are saved in a glossary and can be used in current and future character extraction processes. Similar to other automated information extraction systems, software that extracts character information requires external knowledge to extract target information. This external knowledge may come from training examples (e.g. expert annotated examples for the software to follow) as employed in Tang and Heidorn [22], extraction rules defined by users, such as for PaleoDeepDive [32], or the application of glossaries or ontologies [19, 20]. CharaParser was designed to extract character information and build domain glossaries simultaneously. Being domain experts, users are familiar with term usages and are capable of categorizing the terms with confidence, especially when source sentences and other contextual information are made available to them. Furthermore, categorical glossaries are reusable knowledge that will benefit other natural language processing applications in the biodiversity domain and are valuable resources for constructing phenotype ontologies. In contrast, the utility of training examples and extraction rules are often limited to the taxon groups or description collections for which they were created [33, 34]. CharaParser also differs from other information extraction systems in that it comprehensively extracts all characters found in a description, not just a predefined set of characters, making it more suitable for generating taxon-character matrices from morphological descriptions.

Other biodiversity information extraction systems, including those extracting taxon names, are reviewed in Thessen, Cui, and Mozzherin [34]. Information extraction systems for biomedical domains that extract gene mentions, protein-protein interactions, etc. are reported and reviewed by the BioCreative workshops [14–17]. Work on information extraction from medical records is also fruitful, for example, in [35, 36]. Related work in computer vision and image processing algorithms has extracted characters from high resolution images of organisms, for example, in [37, 38]. While automatic identification of taxa has been called for [39], training computers to score characters from images is challenging as algorithms have to be crafted to extract different types of characters and the target characters may be clustered with other (non)characters in images.

In this paper, we introduce the Explorer of Taxon Concepts (ETC) toolkit, which has been developed in the Exploring Taxon Concepts project to offer a suite of web-based software tools for morphological character extraction, taxon-character matrix building, interactive taxonomic key generation, and taxonomic concept analyses. The tools attempt to work towards a number of challenges, especially on open, computable data, and advance understanding of the evolution of taxonomic names. The toolkit currently consists of Text Capture (powered by CharaParser [29]), Ontology Building (in development), Matrix Generation, Key Generation, and Taxonomy Comparison (powered by Euler [1]) tools, in addition to supporting functionalities for input file creation, file management, task management, and user account management. The tools expect input in English. The tools can be used individually or collectively as a pipeline. We share the belief of Hardisty and Roberts [40] for projects to “release their service early and update often, in response to user feedback”. The ETC tools are made available when implemented and are updated frequently with new or improved features. The ETC Toolkit is currently in beta test and publicly available at http://etc.cs.umb.edu/etcsite/. A different development site is used internally to test new functions and conduct evaluation experiments, including the one reported in this paper.

Taxon-character matrices produced by ETC tools are raw matrices as the character states (i.e., values of the characters) are extracted from taxonomic descriptions without being refined (e.g., as described in [41]) or scored. Some phylogenetic analysis software [2, 3] requires that character states be scored, that is, to convert the raw values to symbols such as 0, 1, and 2, where 0 = small, 1 = medium, and 2 = large, for example. Matrix Converter [42], an open source program software, can be used to score ETC raw matrices to phylogenetic matrices. ETC Matrix Generation allows characters described at a higher rank (e.g. family) to be automatically propagated to lower ranks (e.g. genera) when the characters are missing at the lower ranks. It also supports inferred presence and absence characters for structures (i.e. organs/parts) similar to [43].

ETC is the first set of tools, to our knowledge, that converts morphological descriptions to taxon-character matrices. The main differences between this tool and other information extraction systems are:

1. It does not have a set of pre-defined target characters to be extracted; rather, it is designed to parse and discover all characters described in input descriptions. Given the variety of biodiversity descriptions, the system cannot predict the characters it may encounter.
2. It targets organism morphological descriptions, but is not limited to any taxon groups.
3. It takes an unsupervised learning approach to extract characters so its extraction targets are not limited to those included in the training examples and the users do not need to provide training examples.
4. It consolidates extracted characters to a taxon-character matrix, allowing character inheritance from higher to lower taxa and absence/presence character reasoning.
5. Because of (1), it also outputs reusable knowledge entities such, as categorical glossaries/ontologies (e.g. using the Ontology Building tool not discussed in this paper).

Although still under continued enhancement, ETC Text Capture and Matrix Generation tools are used by a small number of biologists to generate matrices or compare taxonomic concepts for systematics and evolutionary research [1]. In this paper, we introduce these two tools through a case study, in which body part measurements from spider taxonomic descriptions are extracted and consolidated as a taxon-character matrix. We compare matrices generated from original and normalized inputs to a hand-curated gold standard matrix. The spider work is an appropriate case study for three reasons. First, the gold standard matrix has been manually curated by experts from the same set of descriptions prior to this study and is used in actual biological research [44–46]. Second, the spider work provides a relatively straightforward numerical measurement extraction task that allows us to focus the discussion on a set of common issues with automated character extraction, leaving the challenges of matrix making with categorical characters for a future paper. Third, the spider case study permits a comparison experiment design that illustrates significant improvements normalized descriptions could bring to the resulting matrix. It also provides an opportunity to discuss steps that authors of taxonomic descriptions can take to prevent some extraction issues.

The paper is organized as follows: ETC tools used in this case study are first described, followed by the experimental design, material preparation, matrix generation procedure, and evaluation metrics in the Methods section. We report the comparison results in the Results section and analyze the differences in the Discussion section, where we also discuss sources of errors and potential solutions. The paper concludes with future development plans.

## ETC tools for matrix generation

**Fig. 1:**
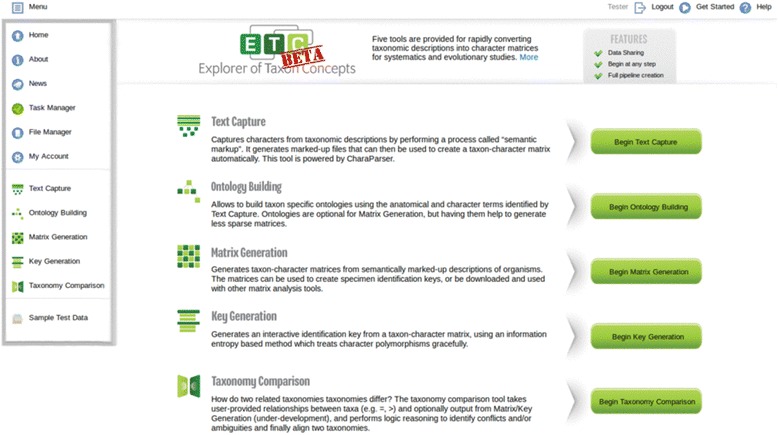
ETC site homepage with expanded menu

The ETC toolkit site (Fig. 1) hosts a set of five tools: Text Capture, Ontology Building, Matrix Generation, Key Generation, and Taxonomy Comparison. These tools are supported by utilities including Task Manager, File Manager, and user account settings. The site Menu, along with Login/out, Get Started, and Help functions, is always available at the top of the screen regardless of the user’s current location. Hovering the mouse over the Menu will provide access to all functions and tools provided by the site.

**Fig. 2:**
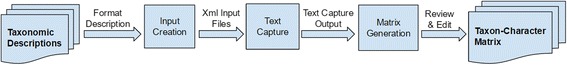
ETC logic flow for generating matrix

ETC toolkit users can utilize the Text Capture and Matrix Generation tools to create taxon-character matrices from taxonomic descriptions. A high level logic flow of the matrix generation pipeline is displayed in Fig. 2.

### Input creation

**Fig. 3:**
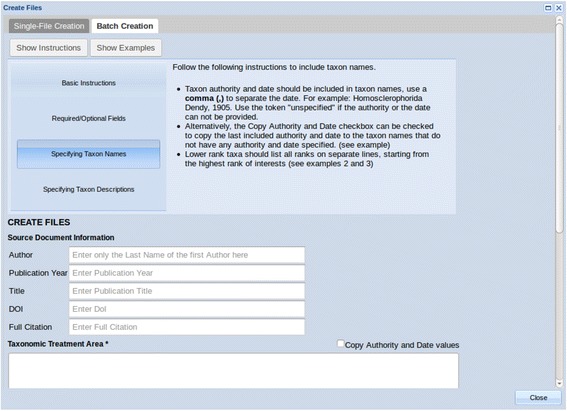
Batch file creation function

Since ETC Text Capture is only concerned with morphological descriptions, it does not directly accept full articles as input. The descriptions to be processed should be manually selected by the user from source publications, and the input files for Text Capture may then be created in the File Manager before starting the tool or within the first step (Create Input) of running the tool, using the Single-File Creation (to create one file at a time) or the Batch Creation (to create multiple files at once) tab. Either tab provides users with a form to enter bibliographic information and paste in plain text taxonomic descriptions, but in Batch Creation, users can format multiple descriptions according to the instructions and paste them into the Taxonomic Treatment Area section to generate multiple input files (Fig. 3).

**Fig. 4:**
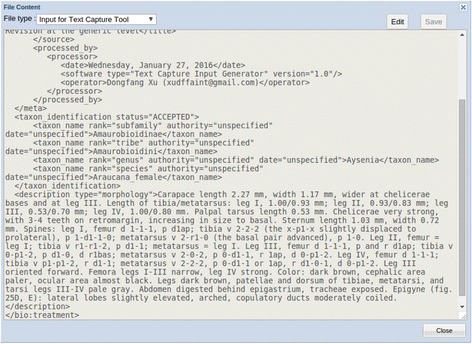
An example input file for the text capture tool

The Input Creation function wraps bibliographic information and descriptions with XML (Extensible Markup Language) tags [47] required by the Text Capture tool. Input files are saved in the File Manager, where the content of a file may be viewed/edited, as shown in Fig. 4.

### Text capture tool

The Text Capture tool employs seven steps to process taxonomic descriptions: Create Input (mentioned above), Define Task, and Preprocess are the preparation steps; Learn, Review, and Parse are processing steps; and Output is the last step (Fig. 9). The Learn and Parse steps are computationally intensive (for algorithmic details, see [29]), while the Review step is where the user interacts with the system to review and categorize character-related terms for the system. These categorizations are reusable for future tasks. In a description, the Text Capture tool annotates structures (labelled as “biological_entity”), characters, character values, and relationships among structures. Figure 9 shows an example of an output file with detailed annotations in XML, conforming to the ETC output schema [48]. These annotations are used by the Matrix Generation tool to produce a matrix. By comparing the XML input (Fig. 4) and the output (Fig. 9), one can see the Text Capture tool breaks down the text descriptions into a series of characters marked up in XML (Note: reading the XML file details is inconsequential to understanding the remainder of the paper).

**Fig. 5:**
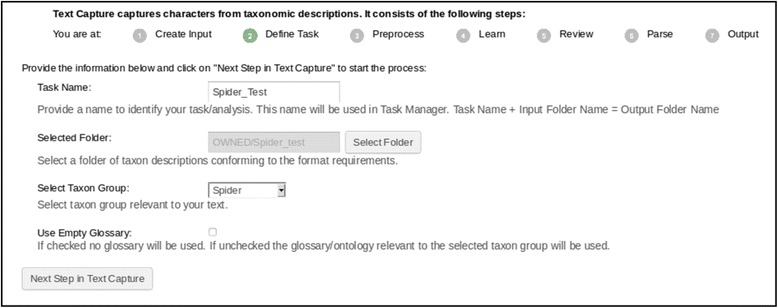
The define task step in the text capture tool

**Fig. 6:**
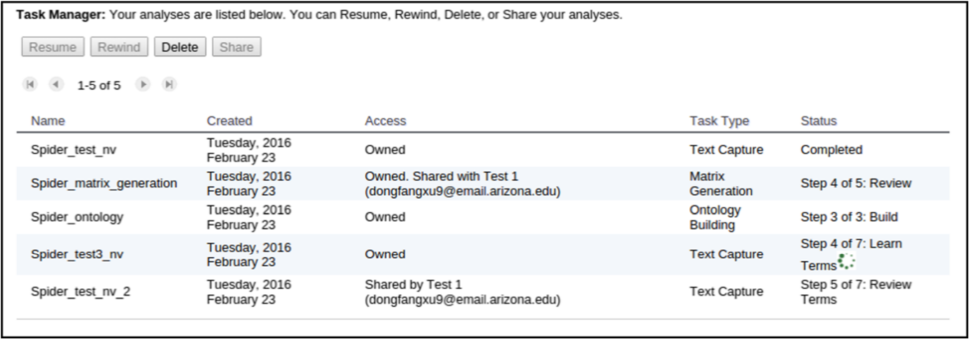
ETC task manager. Shown five tasks with their names (Name), task start time (Created), how the tasks are shared (Access), type of tasks (Task Type), and task progress/current step (Status). The *green* spinning wheel indicates the task is currently running at a specific step

Users start a Text Capture task by generating input files (Create Input, described above) and then define the task by assigning it a name and selecting appropriate settings (Define Task, Fig. 5). The task name is used to name output folders and track the progress of the task. Tasks may be performed asynchronously and users are notified via email or in the Task Manager when the task is completed (see the Status column in Fig. 6, a spinning wheel indicates the task is currently running). The Task Manager can also be used to delete a task or share a task with other registered users. When a task is shared with other users, these users can access the shared task as well as its input and output files. For example, users with a shared Text Capture task can categorize the same set of terms in the Review step and share their expertise with the task owner.

A task is defined with its name, input folder, and the taxon group most closely related to the task (Fig. 5). The taxon group information allows the system to select an appropriate categorical glossary to use to process the task. Currently, there are glossaries for Algae, Cnidaria, Fossil, Gastropods, Hymenoptera, Plant, Porifera, and Spiders (made for the spider case study), reflecting the taxon groups that have been processed with ETC. Using existing glossaries reduces user effort during the Review step. If ETC does not have a needed glossary, the Use Empty Glossary option will make Text Capture learn the terminology from scratch. With the user’s permission, categorizations are exported to OTO (Ontology Term Organizer) [28], reviewed by multiple domain experts, integrated into ETC categorical glossaries, and made publicly available at the ETC Glossary Git Repository [49]. The user’s ownership of the terms set and its categorizations is acknowledged in OTO and in the final glossary. The linkage of the ETC Text Capture tool to OTO allows the collective and incremental building of consensus-based domain controlled vocabularies from source descriptions.

**Fig. 7:**
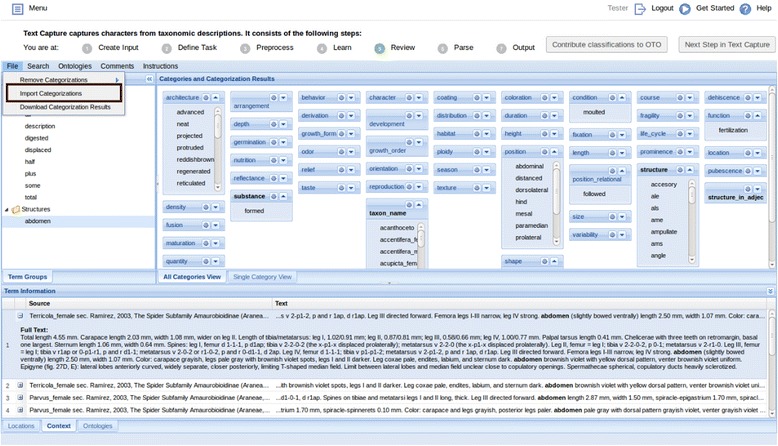
The review step in the text capture tool

The Preprocess step checks for editing errors in the input description text for the user to correct (i.e., unmatched brackets). In the Learn step, the tool analyzes input descriptions and categorizes terms as structures, characters/states, and other terms. With the built-in categorical glossary, it further categorizes character/state terms (e.g., round, large) to specific categories, such as shape and size. Users are then presented with a screen in the Review step to examine system categorizations and categorize remaining terms (Fig. 7). Numerical values are handled automatically by the software and are not presented for review.

**Fig. 8:**
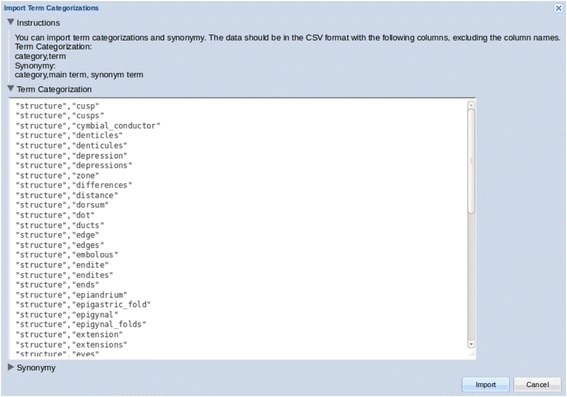
Importing term categorizations in the text capture review step

In the Review interface (Fig. 7), terms to be categorized appear in the pane on the left, while categorized terms are in the category boxes on the right. To categorize a term, the user can drag and drop the term on the heading of its proper category, or use the dropdown menu invoked by right-clicking on the term. The user can indicate two terms are synonyms by dropping one on top of the other, making the latter a primary term (called “preferred term” or “label” in controlled vocabularies or ontologies). This signals the software to replace occurrences of the synonyms with the primary term in the output, making the matrix result cleaner and less sparse. Terms that are neither structures nor characters/states should be left uncategorized in the left pane. The categories are used in Text Capture tool as a flat list of categories without hierarchical relationships. Several user-friendly features are provided: users can add/remove/merge categories, multiple terms can be selected and categorized to one category as a batch, missing terms can be added, misspelled terms can be corrected, terms can be marked as “not useful” and grayed out, and comments can be left on a term. The Term Information pane located at the lower portion of the screen provides useful information about a term. Clicking on a term displays its current categories in the Location tab, its source sentences in the Context tab, and its matching entries from user selected ontologies in the Ontologies tab. The user can make their ontology selections from NCBO BioPortal [50] using the Ontologies menu. The File menu allows users to download their categorization results or upload a set of categorized terms to the system (Fig. 8).

The Review stage is the only step where substantial user input is needed. Term categorizations affect the formulation of characters in the final matrix. For example, if *long* is categorized as a *shape*, the matrix will contain characters such as “leg *shape*: long”, as opposed to *long* as a *size*, which results in “leg *size*: long”. In the spider case study presented in this paper, our focus was on *numerical* measurements; numerical values do not need to be categorized by users, thus, the importance of the Review step is minimized in this particular case.

**Fig. 9:**
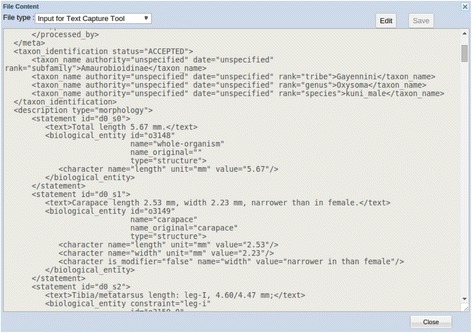
Example output file of the text capture tool

After the Review step, the system parses the descriptions and produces valid XML files (Fig. 9) with structure and character information finely marked up according to the ETC output schema [48]. This schema was developed because existing XML schemas, such as TaxPub [51] or NeXML [52] cannot accommodate the fine-grained markup produced by Text Capture. Furthermore, the XML schemas used in ETC are intended for internal use only, not for data exchange among different systems.

### Matrix generation tool

The output XML files from Text Capture are used as input for the Matrix Generation tool, which consolidates the annotated character information into a taxon-character matrix for users to review and edit (Figs. 11, 12 and 13). The matrix output is a CSV (comma separated values) file (Fig. 14).

Matrix Generation consists of five steps: Select Input, Define Task, Generate, Review, and Output, with Generate being the key processing step.

**Fig. 10:**
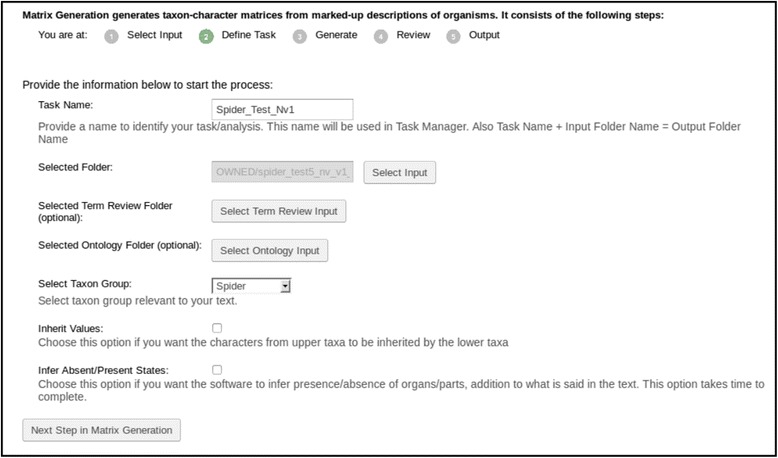
The define task step in the matrix generation tool

The Select Input and Define Tasks steps (Fig. 10) serve similar functions as the first two steps in Text Capture. The Inherit Values enables automatic propagation of characters from a higher taxon to lower taxon members. The Infer Absent/Present States option enables the software to infer the presence or absence of organs and other anatomical parts, aside from what is stated explicitly within the descriptions. For example, if a *leg cusple* is present, then the *leg* must be present, as the Spider Ontology [53] indicates *leg cusple* is part of some *leg article* and *leg article* is part of some *leg*.

The Generate step extracts character information from XML files and assembles them into a taxon-character matrix. The matrix will contain more characters/states than those explicitly stated in the descriptions when the Inherit and/or Infer option is selected.

**Fig. 11:**
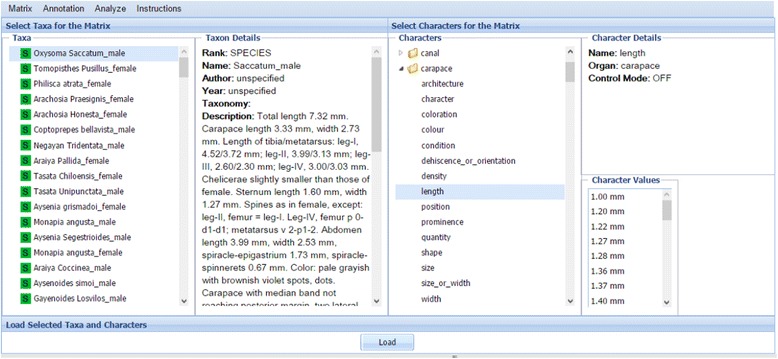
The preview and selection view at the matrix review step

**Fig. 12:**
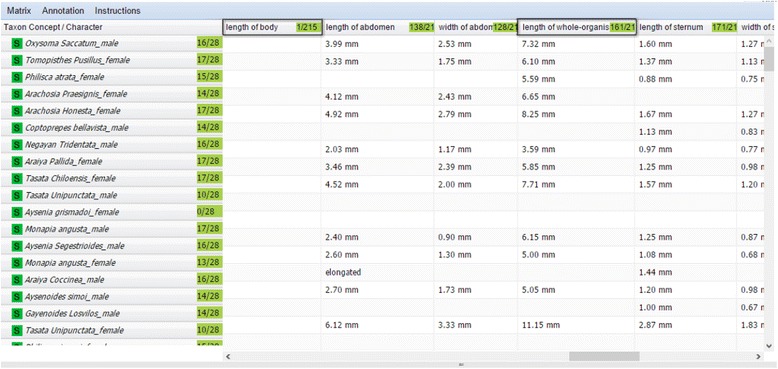
The spreadsheet view at the matrix review step. Two highlighted characters are candidates for a merge

After the matrix is produced, users proceed to the Review step, which has two views: the Preview and Selection View and Spreadsheet View. The Preview and Selection View provides an overview of the taxa and characters produced and allows users to select a set to upload and review (Fig. 11). The Spreadsheet View presents the selected taxa and characters in a matrix format with taxa displayed as rows and characters as columns (Fig. 12).

Web browsers cannot handle large spreadsheets efficiently, therefore it is recommended to review in sections matrices larger than 500 taxa and 500 characters. The selection feature is also useful when the user is interested in only a portion of extracted characters, for example, numerical characters in the spider case study. In the Preview and Selection view (Fig. 11), users are presented with an interface where the left half of the screen is dedicated to information about taxa and the right half to character information. The leftmost panel allows users to select taxa of interest. By right-clicking on each taxon, users may add, modify, remove, or change the order of taxa, or query Google Images to retrieve images of the selected taxon. The Taxon Details panel provides information about the selected taxon, including publication information and the original description. Characters are selected from the Characters panel (the second from right). In the Characters panel, organs and characters are organized in a tree format, with characters listed under their respective organs. Similar to the functions for taxa, users may add, modify, and remove characters or change the order by right-clicking on a character. Details about characters are presented in the upper right panel and character values are displayed in the lower right. The Annotation menu supports user comment and color setting configuration for better data management. Users can associate different colors with different meanings and use colors to track characters that are reviewed, questionable, or require additional research, etc.

**Fig. 13:**
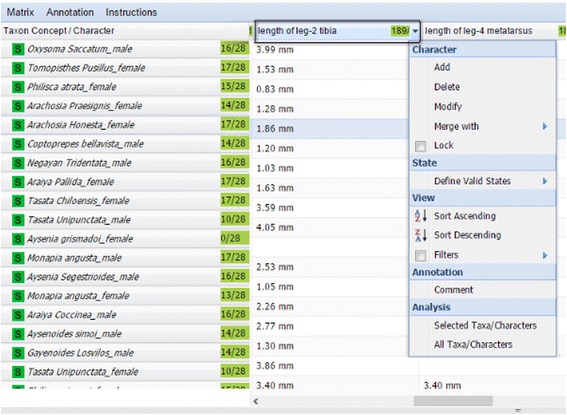
Character-related functions for the first column of the spreadsheet

Multiple taxa and characters can be selected by pressing the “shift” key or “ctrl” key (“shift” or “command” key on a Mac) and *load t*o the Spreadsheet View (Fig. 12). This view provides rich functions that are invoked by hovering the cursor over the right-end of a cell and clicking on the small triangle icon that appears. Functions affecting both taxa and characters are invoked in the first cell of the spreadsheet, taxon-related functions are invoked in any taxon cell (first column in the spreadsheet), and character related functions (Fig. 13) are invoked in any character cell (first row in the spreadsheet).

**Fig. 14:**
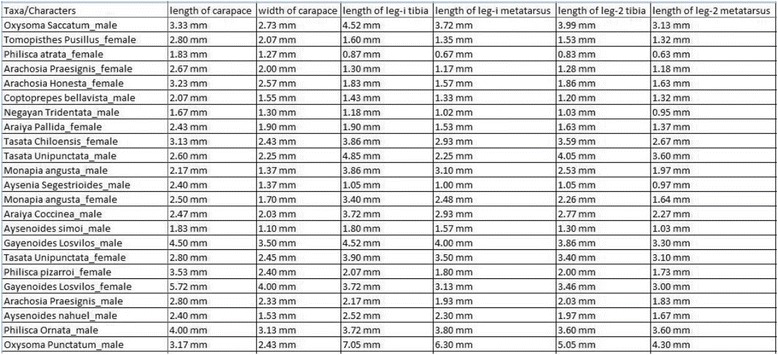
Example output (CSV) file of the matrix generation tool

In addition to the add, delete, edit, sort, color, and comment functions, the user can lock taxa or characters to prevent edits, bring up an original source description, control what values are acceptable for certain characters, or merge two selected characters. Because synonyms or quasi-synonyms are often used, some characters could be merged to consolidate the taxa-character matrix. For example, in Fig. 12 *the length of whole-organism* and *length of body* characters both represent the total length of a spider. The merge function put the values of the two characters in the column the user chooses. After an edit operation is performed, the matrix is refreshed automatically and affected cells are indicated with small red triangles on a corner. Users can save their progress and return to it at a later time via Task Manager. The matrix can also be downloaded from the Matrix menu at any time. When proceeding to the Output step, the matrix is saved as a CSV file (Fig. 14) in the File Manager.

It is important to note that the basic principle for designing Text Capture and Matrix Generation tools is to stay true to the original descriptions. Taxonomic descriptions are highly technical and present levels of sophistication and subtleties that only taxon experts can fully interpret. The interpretation may also depend on the intended use of the character data. The ETC tools are intended to extract characters as they are expressed in taxon descriptions but leave the customization and refinement of the results to the experts, which may be accomplished by using the functions provided at the View step. In addition, since parsing characters and generating matrices are automated using various algorithms, the results are not error free. The rest of the paper evaluates the performance of the ETC matrix generation pipeline using the spider body part measurement as a case study.

## Methods

### Experimental design

The experiment was designed as a comparison study. The same ETC tools and same settings were applied to the set of original input descriptions and the normalized set, then machine-generated matrices were compared to a gold standard matrix. Both versions of the input descriptions were generated from the same source publications on spiders (see the Materials section). The hypothesis was that the normalized input would result in a taxon-character matrix that is more similar to the gold standard than when using the original input. The original input and the normalized input used in the experiment are included in the Additional file 1 as Original Input and Additional file 2 as Normalized Input.

The Gold Standard matrix was built by the three spider systematists who co-authored the paper. The building of the gold standard matrix was blind to all the other co-authors. Two other co-authors normalized the text, and one of them generated the matrices using ETC tools.

### Materials

The gold standard matrix was derived from a matrix initially composed in Ramírez [44] and successively enriched/used in Aisen and Ramírez [45] and Labarque et al. [46]. This expert-curated matrix contained 234 descriptions of exemplars (an exemplar is a male or a female of a given species) of 122 species (not all species have both exemplars known) and 21 characters. To create the gold standard matrix for this study, 26 species with no descriptions or with non-English descriptions and three redundant characters were removed from the expert matrix. In addition, character names in the expert matrix were standardized to the *character of structure* style (e.g., “length of tibia”), making them comparable to machine-generated character names. When this matrix was used to evaluate the machine-generated matrices, a handful of incorrect states/values were found and subsequently corrected. The final gold standard matrix consisted of 188 exemplars of 96 species, 18 characters and 2921 character states. The gold standard matrix is included in the Additional file 3 as Gold Standard Matrix.

All 799 terms representing anatomical parts in the Spider Ontology [53] and 6970 terms representing character/character states from the Plant Glossary [54] were used to create a new categorical glossary for spiders. Although the character/character state terms from the Plant Glossary do not affect the machine-generated numerical measurement matrices, they were included to reduce the number of terms for review/categorization in Text Capture. The spider glossary files, one for term categorization and the other for synonyms, can be found at: https://github.com/biosemantics/glossaries/tree/master/Spider.

Two versions of the input descriptions, original and normalized, were generated from the source publications provided by the spider systematist co-authors. Content of male and female exemplar descriptions of the spider species were manually copied from PDF (Portable Document Format) files and formatted as required by the ETC Input Creation utility. Errors in pasted text were manually corrected (e.g., an “=” being pasted as a “5”), and different characters appearing as hyphens (“-”) were converted to standard ASCII (American Standard Code for Information Interchange) hyphens. The resulting clean text descriptions were considered as the *original* input. A *normalized version* was formed by adding omitted measurement units (i.e., “mm”) and the omitted word “leg” to the original input. For example, an original statement “tibia/metatarsus: I, 0.42/0.32” was normalized to “tibia/metatarsus:*leg* I, 0.42/0.32 *mm*”. Two of the co-authors created the normalized input programmatically and independently cross-validated the results. While normalization is a typical step in text processing, the specific normalization step taken in this experiment was meant to put the missing information (units and legs) back to the text to make the descriptions more self-contained. In the results we examine the different performance results from using the self-contained (normalized) vs. the original descriptions. The automatic modification of text method used in [55] (replacing “…” with real drug names) achieved the same goal of making the text self-contained.

### Generation of the matrices using software tools

The Input Creation, Text Capture, and Matrix Generation tools on the ETC-development site were used as a pipeline to generate a matrix for each of the two input texts. For the Text Capture tool, the “Spider” glossary was used (Fig. 5). Terms pertaining to structures were mostly categorized correctly by the software while some structure terms, such as “coxae” and “membrane”, were manually categorized (Fig. 7). Because this study was primarily concerned with the numerical measurements of morphological structures, categorical character terms (e.g., *present*, *robust*) were placed in an arbitrary category (“architecture”) and essentially ignored. No synonyms were made in the Review step. Term categorizations made in this experiment is included in the Additional file 4 as Term Categorization. In the Matrix Generation tool, the Inherit Values and Infer Absent/Present States options were not selected (Fig. 10) because they were not relevant for this case study. After morphological characters were extracted by the Matrix Generation tool, body part characters with numerical measurements were manually selected in the Preview and Selection View to form the final output matrix (Fig. 14).

### Comparing ETC matrices to the gold standard matrix

To evaluate the effectiveness of the Matrix Generation workflow, we measured the accuracy of the extracted characters, the effort needed to edit the matrices, and the similarity of the edited matrices to the gold standard matrix.

Accuracy measures the correctness of extracted data items. An extracted data item is correct if it is consistent with the original description. A data item may be considered correct while not matching the gold standard as the latter represents the expert’s consolidation and refinement of original characters present in the descriptions. For example, if the system extracts *length of ii = 1.35 mm* from the description *I, II, III, IV length: 1.23, 1.35, 1.27, 1.28 mm*, it will be considered correct, even though the gold standard may have *length of leg ii = 1.35 mm*. However, if the system extracts *size of ii = 1.35 mm, length of ii = 1.35(missing unit), or length of ii = 1.27 mm*, it would be considered wrong.

$$ \mathrm{accuracy} = \left|\mathrm{correct}\ \mathrm{data}\ \mathrm{items}\right|\ /\ \left|\mathrm{extracted}\ \mathrm{data}\ \mathrm{items}\right| $$

Edit effort is the minimal number of *column-based* merge, rename, and deletion operations needed to make a machine-generated matrix as similar as possible to the gold standard matrix. Specifically, character columns mostly containing incorrect values (i.e. values not present in the gold standard) are deleted; and character columns mostly containing correct values are either merged into a valid column/character (i.e., matching characters present in the gold standard) or renamed with a valid column/character name. Although merge operations place character values under a different character name, they are not counted again as rename operations. We also note that some characters extracted by the machine need splitting to match the gold standard, for example, *length of tibia/metatarsus = 0.52/0.44 mm* needs to be split into *length of tibia = 0.52 mm* and *length of metatarsus = 0.44 mm*. Since a split character feature has not been implemented in the matrix review step, in this study, split operations were counted separately from edit effort. Edit efforts were counted manually. Edits made on machine-generated matrices are documented in Matrix Edits in the Additional file 5.

The similarity of an edited matrix to the gold standard is evaluated via precision, recall, and F1 metrics that are routinely used in the evaluation of information retrieval and information extraction systems. Precision is the proportion of machine-generated data items that match the gold standard. Recall is the proportion of gold standard data items that are extracted by the machine. In other words, precision measures the soundness of machine-generated items, while recall measures the exhaustiveness of the machine results as compared to the gold standard. Both metrics result in a value between 0 and 1. If the machine-generated matrix is identical to the gold standard, both precision and recall will have a value of 1. The F1 score is the harmonic mean of precision and recall, meaning it puts equal weights to precision and recall. To summarize:

- Precision = |matched data items| / |data items output by the software, excluding null values|
- Recall = |matched data items| / |data items in the gold standard matrix, including null values|
- F1 score = 2 * Precision * Recall / (Precision + Recall)

Note that accuracy is based on the correctness of the extracted characters as compared to the input text, while precision is based on the “matchness” of the extracted characters to the gold standard.

## Results

Table 1 describes the matrices generated from the original and normalized inputs, before and after the edits. For all matrices, the number of rows is 188, consistent with the input exemplars. The matrix from the original input initially had 41 characters and 2942 non-empty data items, making the matrix 38.17% populated, while the matrix from the normalized input initially had 43 characters and 2914 non-empty data items, or 36.05% populated. After the edits, both matrices were better populated at approximately 85%.

**Table 1:** Summary of the matrices generated from the original and the normalized inputs

	Original input matrix		Normalized input matrix	
	Before edits	After edits	Before edits	After edits
Rows/Exemplars	188	188	188	188
Columns/Characters	41	18	43	18
Non-empty cells	2942	2864	2914	2913
Fullness of matrix (%)	38.17% populated	85.00% populated	36.05% populated	86.08% populated

Table 2 below summarizes the accuracy, edit effort, and precision/recall-based similarity scores for the matrices generated from the original input and the normalized input. The pre-edit accuracy of the matrix from the original input is 1.46%, and after 46 column-based edits, its precision, recall, and F1 scores were 99.79, 98.82, and 99.35% respectively. The pre-edit accuracy of the matrix from the normalized input was 98.83%, and after 28 edits, its precision, recall, and F1 scores were 99.91, 99.65, and 99.78%, respectively. The pre-edit accuracy of normalized input matrix was 67 times better than that of the original input matrix, and normalized input matrix required 39% less edit effort.

**Table 2:** Accuracy, edit effort, and precision/recall/F1-based similarity scores of the matrices generated from the original and normalized inputs

	Original input matrix	Normalized input matrix
Pre-edit accuracy (%)	1.46% = 43/2942	98.83% = 2880/2914
Number of edits (splits)	46 (8)	28 (3)
Post-edit precision (%)	99.79%	99.91%
Post-edit recall (%)	98.92%	99.65%
Post-edit F1 score (%)	99.35%	99.78%

Table 3 summarizes the edits made to the two matrices. Details are included in Tables 4 and 5. For the matrix resulting from the original input, eight of the 41 characters were first split because they contained data items with combined character values. After that, 46 edits (=9 characters (i.e., columns) deleted + 15 characters renamed + 22 characters merged) were made on the matrix. These edits affected 3593 values in the matrix. Three characters out of the original 41 were retained without edits.

**Table 3:** Summary of edit efforts made to the original and normalized input matrices

	Original input matrix	Normalized input matrix
Splits	8	3
Deletions	9	3
Renames	15	0
Merges	22	25
Unedited	3	18
Values affected by edits	3593	162

**Table 4:** Edit operations performed in the matrix generated from the original input

Edit type	Characters affected	Operations	Edit effort
Delete	quantity of leg [15], character of carapace [4], length of carapace [147], width of carapace [76], length of abdomen [133], length of sternum [168], quantity of iii tibia/metatarsu [1], quantity of iii [8], quantity of leg-3 [1]	delete column	9
Rename	quantity of whole-organism [162]	Rename as "length of whole-organism (new)"	1
	quantity of carapace (split, length) [139]	Rename as "length of carapace (new)"	1
	quantity of carapace (split, width) [129]	Rename as "width of carapace (new)"	1
	quantity of abdomen (split, length) [133]	Rename as "length of abdomen (new)"	1
	quantity of sternum (split, length) [165]	Rename as "length of sternum (new)"	1
	quantity of spiracle-epigastrium [138]	Rename as "distance of spiracle-epigastrium (new)"	1
	quantity of spiracle-spinneret [155]	Rename as "distance of spiracle-spinneret (new)"	1
	quantity of i tibia [189]	Rename as "length of leg i tibia (new)"	1
	quantity of i metatarsus [189]	Rename as "length of leg i metatarsus (new)"	1
	quantity of ii tibia [188]	Rename as "length of leg ii tibia (new)"	1
	quantity of ii metatarsus [188]	Rename as "length of leg ii metatarsus (new)"	1
	quantity of iii tibia [185]	Rename as "length of leg iii tibia (new)"	1
	quantity of iii metatarsus [185]	Rename as "length of leg iii metatarsus (new)"	1
	quantity of iv tibia [186]	Rename as "length of leg iv tibia (new)"	1
	quantity of iv metatarsus [186]	Rename as "length of leg iv metatarsus (new)"	1
Merge	length of whole-organism (new) [162], quantity of body [1]	Merge into length of whole-organism (new)"	1
	length of carapace(new) [129], quantity of prosoma(split, length) [37], quantity of thoracic-groove [6], quantity of cephalic-area [1], quantity of front [2], quantity of ocular-area(split, length) [2]	Merge into “length of carapace (new)”	5
	width of carapace (new) [139], quantity of prosoma(split, width) [37], quantity of ocular-area(split, width) [1]	Merge into “width of carapace (new)”	2
	length of palpal-tarsus [5]*, quantity of palpal-tarsus [57]	Merge into “length of palpal-tarsus”	1
	length of abdomen (new) [133], quantity of opisthosomum(split, length) [44]	Merge into “length of abdomen (new)”	1
	width of abdomen [2]*, quantity of opisthosomum(split, width) [7], quantity of abdomen (split, width) [128]	Merge into “width of abdomen”	2
	quantity of sternum (split, width) [160], width of sternum [1]*	Merge into “width of sternum”	1
	distance of spiracle-spinneret (new) [155], quantity of spiracle [1], quantity of spiracle spinneret [2]	Merge into “distance of spinneret-spiracle(new)”	2
	quantity of epigastric-furrow [1], distance of spiracle-epigastrium (new) [138], quantity of epigastrium-epigastrium [1], quantity of epigastrium-spiracle [20]	Merge into “distance of epigastrium-spiracle (new)”	3
	length of leg ii tibia (new) [188], quantity of ii (split, tibia) [5]	Merge into ”length of leg ii tibia(new)“	1
	length of leg ii metatarsus (new) [188], quantity of ii (split, metatarsus) [3]	Merge into “length of leg ii metatarsus(new)”	1
	length of leg iv tibia (new) [186], quantity of iv (split, tibia) [4]	Merge into "length of leg iv tibia(new)"	1
	length of leg iv metatarsus (new) [186], quantity of iv (split, metatarsus) [3]	Merge into "length of leg iv metatarsus(new)"	1
Total edits	46		

The numbers in “[]” indicate the number of values affected by an edit operation. Characters indicated with an “*” were retained without edits

**Table 5:** Edit operations performed in the matrix generated from the normalized input

Edit type	Characters affected	Operation	Edit effort
Merge	1. length of whole-organism [161], length of body^$1^ [1]	Merge into *length of whole-organism*	1
	2. length of carapace [147], size of carapace [4], length of prosoma^$2^ [37], length of ocular-area [1], length of thoracic-groove [2], length of cephalic-area [1]	Merge into *length of carapace*	5
	3. width of carapace [152], width of prosoma^$3^ [37], width of ocular-area [1], width of thoracic-groove [2], width of cephalic-area [1]	Merge into *width of carapace*	4
	4. length of abdomen [138], length of opisthosomum^$4^ [44]	Merge into *length of abdomen*	1
	5. width of abdomen [128], width of opisthosomum^$5^ [6]	Merge into *width of abdomen*	1
	6. location of spiracle [1], size of spiracle spinneret [2], distance of spinneret-spiracle [155]	Merge into *distance of spinneret-spiracle*	2
	7. distance of epigastric-furrow [1], distance of epigastrium-epigastrium^$6^ [1], distance of epigastrium-spiracle [158]	Merge into *distance of epigastrium-spiracle*	2
	8. length of leg-2 tibia [189], length of leg-2 [1], size_or_shape of leg-2 (split, tibia) [2]	Merge into *length of leg-2 tibia*	2
	9. length of leg-2 metatarsus [189], size_or_shape of leg-2 (split, metatarsus) [2]	Merge into *length of leg-2 metatarsus*	1
	10. length of leg-iii tibia [186], length of leg-ii [1]i, size_or_shape of leg-iii (split, tibia) [1]	Merge into *length of leg-iii tibia*	2
	11. length of leg-iii metatarsus [186], size_or_shape of leg-iii (split, metatarsus) [1]	Merge into *length of leg-iii metatarsus*	1
	12. length of leg-4 tibia [187], length of leg-4 [1], size_or_shape of leg-4 (split) [2]	Merge into *length of leg-4 tibia*	2
	13. length of leg-4 metatarsus [187], size_or_shape of leg-4 (split) [2]	Merge into *length of leg-4 metatarsus*	1
Delete	14. length of leg [3]	delete *length of leg*	1
	15. size of abdomen [3] (values are non-numerical, e.g. tiny)	delete *size of abdomen*	1
	16. length of leg-iii tibia/metatarsu [1]	delete *length of leg-iii tibia/metatarsu*	1
Total edits			28

The numbers in “[]” indicate the number of values affected by an edit operation. The 18 characters in the gold standard were all included in the machine-generated matrix. The characters superscripted with “$N” are considered equivalent to a corresponding character in the gold standard, either by their semantic equivalence (i.e.. $1), or by the experts’ decisions (i.e., $2–$6)

For the matrix generated from the normalized input, three of the 43 initial characters were split at first, and then 28 edits were made (including three deletions and 25 merges), with a total of 162 values affected by the edits.

Precision, recall, and F1 scores of the edited matrices as compared to the gold standard are shown in Tables 6, 7, 8 and 9. Tables 6 and 7 display the exemplar-based and character-based precision and recall scores of the matrix generated from the original input, while Tables 8 and 9 show the scores of the matrix generated from the normalized input. The scores are very similar between the two matrices. In Table 2, the precision/recall scores of all data items in post-edit matrices from the original and normalized inputs are 99.79%/98.92% and 99.91%/99.65%, respectively. It indicates that 0.21% and 0.09% of the data items in these two matrices are incorrect, respectively, and 1.08% and 0.35% of the data items in the gold matrix were missing from these matrices, respectively.

**Table 6:** Exemplar-based precision, recall, and F1 scores of the matrix generated from the original input

	Mean	Sd	Min	Max	Number
Precision	0.9981	0.01063	0.9333	1	188
recall	0.9805	0.05153	0.7222	1	188
F1 score	0.9886	0.03055	0.8387	1	188

**Table 7:** Character-based precision and recall of the matrix generated from the original input

Character	Precision	Recall	F1-score	Character	Precision	Recall	F1-score
Length of whole-organism	0.9947	0.9894	0.992	distance of spinneret-spiracle	1	1	1
Length of carapace	0.9883	0.8989	0.9415	length of leg-i tibia	1	0.9947	0.9973
Width of carapace	1	0.9149	0.9556	length of leg-i metatarsus	1	0.9947	0.9973
Length of palpal-tarsus	1	1	1	length of leg-ii tibia	1	1	1
Length of sternum	1	0.9681	0.9838	length of leg-ii metatarsus	1	1	1
Width of sternum	1	0.9787	0.9892	length of leg-iii tibia	1	0.9787	0.9892
Length of abdomen	0.9836	0.9574	0.9704	length of leg-iii metatarsus	1	0.9840	0.9920
Width of abdomen	0.9947	0.9894	0.992	length of leg-iv tibia	1	0.9947	0.9973
Distance of epigastrium-spiracle	1	1	1	length of leg-iv metatarsus	1	1	1

**Table 8:** Exemplar-based precision, recall, and F1 scores of the matrix generated from the normalized input

	Mean	Sd	Min	Max	Number
Precision	0.9991	0.00698	0.9444	1	188
Recall	0.9965	0.01872	0.8333	1	188
F1 score	0.9977	0.01158	0.9091	1	188

**Table 9:** Character-based precision, recall, and F1 scores of the matrix generated from the normalized input

Character	Precision	Recall	F1 score	Character	Precision	Recall	F1 score
Length of whole-organism	1	0.9947	0.9973	distance of spinneret-spiracle	1	1	1
Length of carapace	1	0.9947	0.9973	length of leg-i tibia	1	0.9947	0.9973
Width of carapace	0.9947	0.9894	0.992	length of leg-i metatarsus	1	0.9947	0.9973
Length of palpal-tarsus	1	1	1	length of leg-ii tibia	1	1	1
Length of sternum	1	1	1	length of leg-ii metatarsus	1	1	1
Width of sternum	1	0.9947	0.9973	length of leg-iii tibia	1	0.9947	0.9973
Length of abdomen	0.9894	0.9894	0.9894	length of leg-iii metatarsus	1	0.9947	0.9973
Width of abdomen	1	1	1	length of leg-iv tibia	1	0.9947	0.9973
Distance of epigastrium-spiracle	1	1	1	length of leg-iv metatarsus	1	1	1

## Discussion

### Domain conventions and automated character extraction

The accuracy scores of the original input and normalized input matrices were dramatically different, 1.46% vs. 98.83%, respectively (Table 2). The lower accuracy of the data items extracted from the original input can almost be exclusively attributed to its omitting units in measurements (“mm”). Measurement units and other indicators, such as “long”, “length”, and “wide”, are among the clues the software uses to identify measurement characters. When measurement indicators are absent, the software labels the character with a more general label (e.g. “size”) in place of a more specific label of “length” or “width”. When both units and measurement indicator clues are absent, as was the case for the majority of the numerical values in the original input, the software could only name the character with the most general label (“quantity”). These quantity characters account for the difference in accuracy scores, which is also reflected in the rename and merge edits for the matrix (Table 4).

Adding the word “leg” in front of Roman numerals “I”, “II”, “III”, or “IV” in the original input did not affect the accuracy score because other cues in the text can be used in its place (in this case, the categorization of Roman numerals as *structures* at the Term Review step). One can take this as a sign of the system’s robustness, but it should be noted that omitting “leg” made character names for the leg measurements less understandable for the user (e.g., “length of ii”), and introduced term categorizations that are domain specific (e.g., categorizing Roman numerals as structures). We would recommend that future description authors not omit the word “leg”, or other structure terms (e.g., ribs) under similar circumstances.

The performance differences between normalized and original descriptions in terms of accuracy and edit efforts shows less self-contained morphological descriptions present a challenge for automated character extraction. It may be argued that software can be made for users to provide missing information during data processing. This approach is not viable in our task for at least three reasons: (1) It is infeasible to collect all special conventions used in describing biodiversity and new conventions may continue to be introduced (e.g., [41]); (2) accommodating a large number of special conventions useful only for specific domains harms the usability of the software for all users -- it requires all users to be aware of conventions used in other domains to discern the ones that are applicable to the task at hand; (3) it also makes the software difficult to develop or maintain in the long run, especially in the presence of conflicting conventions. To assist with automated character extraction and reduce human effort, we encourage systematics authors to write more self-contained morphological descriptions [56].

Although not included in this experiment, we would like to note that descriptions codified according to certain conventions are difficult to parse automatically for the same reason -- the needed information on how to interpret them is not in the description text. For example, “Spines: leg I, femur *d 1-1-1, p d1ap*; tibia *v 2-2-2* (the x-p1-x displaced to prolateral), *p 1-1-1* or *1-0-1, r 1-0-1*; metatarsus *v 2-0-2, p d1-d1-0-1, r 0-1-0, d 0-p1-0.*”

### Matrix generation pipeline performance

Although both machine generated matrices had become very similar to the gold standard matrix after editing (99% precision and recall, Table 2), the matrix from the original input needed nine deletions, 15 renames, and 22 merges while the matrix from the normalized input needed much fewer edits (three deletions, 0 renames, and 25 merges). The latter edits affected fewer values (see the counts in “[]” in Tables 4 and 5): it was often the case a few values were merged to a correct character already present, suggesting the matrix generated from the normalized input was already quite similar to the gold standard. The merges were sometimes needed for characters that were correctly extracted. For example, *length of abdomen* and *length of opisthosoma* were distinct characters correctly extracted from the original description, but for the specific use of the matrix in [44–46], the spider experts considered them as equivalent characters (note: *opisthosoma* and *abdomen* were not treated as synonyms in the Spider Ontology [53]). This phenomenon confirms our design rationale for the merge operation in the matrix review interface. In addition, the character *distance of epigastrium-epigastrium* (Table 5) was also correct according to the original descriptions, but the experts identified it as a typo in the original publication (should be epigastrium-spiracle).

ETC provides functions to control equivalent character issues, for example, synonymizing terms or importing synonyms in the Review step of Text Capture (Fig. 7), and the ETC Ontology Building tool that is being implemented. True synonyms can be included in the ontology, while terms that need to be treated as synonyms for a certain task may be synonymized in the Review step. Making use of these tools could reduce the effort of merging characters during the Matrix Review step.

The precision and recall scores (Tables 6, 7, 8, and 9) of the exemplar-based and character-based evaluation of edited matrices indicate that character *values* were extracted from the descriptions correctly (high precision) and not many were missed (high recall), even after deletions of some characters. Character/column-based editing seems to be effective in bringing machine-generated matrices close to the gold standard matrix. The low standard deviations of precision and recall scores in Tables 6 and 8 suggest that the software performs consistently on each exemplar and across all characters, as shown by the similar precision and recall scores for each of the characters in Tables 7 and 9.

### Error analyses

Incorrect decisions in character markup made by Text Capture (parse step) propagate into the matrices generated. In this section, we discuss the errors and their causes, which include one caused by a typo in the original source PDF file where “width” was misspelled as “with”.

ISSUE I: There were several mistakes that led to a number of merge operations (e.g., in rows two and three in Table 5). If analyzed carefully, it can be seen the description sentences were ambiguously written. Three such examples are given below:

1. **Carapace** globose, **thoracic groove** on depressed area, length 3.27 mm, width 2.70 mm.
2. **Carapace** very wide in front, **ocular area** slightly protruding, length 2.30 mm, width 1.57 mm.
3. **Abdomen** extremely elongate, **legs** very long, including **leg III.** Total length 7.58 mm.

In each example, the length/width measurements could be associated with any structure shown in bold (a thoracic groove or ocular area could have a length and a width, and leg iii could have a total length). This type of ambiguity is difficult to resolve, even by a non-expert human reader. These sentences can be simply revised as below to remove the ambiguity.

1. (Revised) **Carapace** globose, length 3.27 mm, width 2.70 mm, **thoracic groove** on depressed area.
2. (Revised) **Carapace** very wide in front, length 2.30 mm, width 1.57 mm, **ocular area** slightly protruding.
3. (Revised) Total length 7.58 mm. **Abdomen** extremely elongate, **legs** very long, including **leg III.**

Semantic ambiguity in taxonomic descriptions is a widespread issue, as we have seen it in all taxon groups we have processed. Sometimes a domain expert is not able to interpret a descriptive statement with certainty, however, it is not always easy for description authors to notice the ambiguity.

ISSUE II: Another error was caused by the software’s inability to translate the following sentence to “distance of spiracle-epigastrium” (this is related to the merge operation on row 7, Table 5).

4. Epigastric furrow 0.74 mm from tracheal spiracle.

This translation requires several approximations: epigastric furrow approximates epigastrium, and tracheal spiracle approximates spiracle. As indicated before, such approximations are purposefully excluded from the design goals of the system; however, the software did recognize that some distance is expressed in the sentence.

ISSUE III: Text Capture needs to be improved in its ability to accurately generate individual characters from compound expressions similar to those included in Sentences 5 and 6 below. The problem was alleviated to some extent by the normalizations, reducing the number of splits by over 50% (from eight splits in the original version down to three in normalized version).

5. Length of tibia**/**metatarsus: *leg I*, 0.52**/**0.44 mm; *leg II*, 0.50**/**0.40 mm; *leg III*, 0.24 mm; *leg IV*, 0.40**/**0.30 mm.
6. Tibial lengths and **indices**: *leg I* missing; *leg II*1.73 mm, **7**; *leg III*0.96 mm, **13**; *leg IV*2.02 mm, **7**.

Sentences 5 and 6 are challenging because the characters (e.g., *length of leg i tibia*) and their values (e.g. *0.52 mm*) are separated by other elements and they require information *external* to the text itself for accurate interpretation, for example, knowing the tibia and metatarsus are parts of each leg, knowing the correspondence of multiple characters (e.g., *length* and *index*) to their listed values, and knowing how to deal with exceptions, such as missing values (e.g., Sentence 5, the length value for leg iii metatarsus is missing). While specific rules can be programmed to parse the sentences seen in this experiment, the applicability of the rules to other descriptions is highly questionable, as they could have their own special conventions.

### Error identification

One practical question is how to quickly identify errors, whether in character names or in values, at the Matrix Review step. The names of the characters themselves are good clues, for example *quantity of leg* would seem suspicious to a spider expert. In addition, the Review interface supports different ways of sorting characters. Assuming erroneous characters would have values in fewer taxa, sort by character coverage (i.e., the number of taxa having a value for a character) can help identify bad characters. The original descriptions can also be brought up from the Matrix Review interface, allowing the user to check the original descriptions. An upcoming feature will highlight characters in the original text to facilitate scanning of text by the user.

### Other types of characters

Issues discussed here are also applicable to extracting and matricizing categorical characters. We know that a character consists of a structure name (e.g., leg), a character name (e.g., length) and a character value (e.g., 0.7 mm). ISSUE I discussed in Error Analysis will affect whether a correct structure name is identified, regardless of types of characters. ISSUE II and III are similarly applicable to categorical characters.

A major challenge specific to categorical characters is with the determination of the character names. Descriptions often state character values without explicitly mention character names. For example, in ocular area slightly protruding (Example 2 in Error Analysis), protruding is the character value, but what is its character? Does protruding describe the size, orientation, prominence, or relief of the ocular area? Character names are important because they determine how the characters will be named in the matrix (e.g., size/orientation/prominence/relief of ocular area). Since a standard character dictionary does not exist, the system has to ask the user to indicate what character name protruding refers to in the Term Review step (Fig. 7). In fact, Term Review could be the most time consuming step when dealing with categorical characters because the user will need to categorize the terms that are not in the system’s glossary. Synonymizing structure/organ names at this step is also critical for producing high quality matrices to avoid the same characters being scattered in multiple columns.

Cardinal characters, such as counts or quantities, are often easier to extract. Our experience suggests that their extraction accuracy from taxonomic descriptions can be expected at the same level as the numerical measurements reported here. However, when the cardinal characters are not expressed in numbers, but in phrases, such as few, many, and numerous, the semantics of the character values will need human interpretation. While the systematics community has discouraged this practice, it still exists in descriptions.

## Conclusions

In this paper, we introduced the ETC matrix generation pipeline, Input Creation -Text Capture - Matrix Generation, for semi-automatic production of raw taxon-character matrices from morphological descriptions of various taxon groups. This is to our knowledge the first pipeline that produces taxon-character matrices from morphological descriptions. We reported a case study where the tools were used to generate two measurement matrices from the original and normalized descriptions of 188 spider exemplars. The quality of the machine-generated matrices were compared to the hand-curated gold standard matrices, in terms of data extraction accuracy, efforts required to edit a matrix, and the similarity of an edited matrix to the gold standard.

As demonstrated in the paper, ETC matrix generation pipeline is a low-barrier workflow, in that it does not require training examples or user-crafted extraction rules. The inputs required are the minimal necessary requirements to perform the task -- clean text descriptions and domain knowledge in the form of term categorization. As shown in [29] and confirmed in this case study, the character extraction works well on fact-rich, self-contained morphological descriptions with relatively simple syntactic structures. Besides generating taxon-character matrices, evidence from this case study suggests other benefits of using the tools: (1) helping to identify errors in the source descriptions (two cases), (2) helping to identify errors in the human-curated matrix (five cases), and (3) checking for parallelism in the descriptions. These errors can be corrected and the can be data re-harvested using tools/infrastructure such as the one described in [57].

The spider body part measurement experiment provided quantitative support for several findings that we argue are not limited to this case study but are generalizable across character information extraction in biodiversity domains:

1. With full respect for any domain conventions, we showed the conventions that make taxonomic descriptions less self-contained have negative impacts on machine-processed results. The accuracy of the data items (i.e., character/value pairs) extracted was improved from 1.46% using the original input to 98.83% using the normalized input (essentially by adding the missing measurement units).
2. Semantic ambiguity exists in morphological descriptions (also see [58]). It is often not easy for description authors to see the ambiguity. We plan to adapt the CharaParser algorithm to highlight the potential semantic ambiguities in the descriptions for the authors.
3. We also showed that accurately extracted data items may not match exactly with the independently-created gold standard, the ideal result desired by biologists. The matrix from the normalized input contained 98.83% accurate data items, but it still required 28 edits to be 99% similar to the gold standard (188 exemplar × 18 character, 2921 values). The analyses of the experimental results revealed two reasons for this: (a) the differences between character expressions used in the original descriptions and the form of characters the user desires in the matrix, and (b) less-parallel descriptions or user/use preferences sometimes lead to the synonymization of an original character to something close. In addition, as elaborated in [41], selecting and constructing characters for certain biological research is a nontrivial task even for domain experts.

In addition, the case study showed that character/column-based edits were sufficient to bring the matrices 99% similar to the gold matrix. Although this result confirms our experience, additional empirical studies are needed to verify this result.

Future work will further improve the character extraction performance and improve the robustness of the system for various inputs. We also hope to enhance the input functionality by taking HTML, DOC, or PDF files as input. PDF is a challenging format for text processing, but promising software is being developed and tested [59]. This experiment suggested that editing facilities are needed for users to identify, select, merge, split, rename, or delete machine-generated characters. The ETC Matrix Review interface already provided a suite of features in this regard, but additional improvement is needed. Some of the features can be easily added, for example, support for quick splitting of a compound character, or color-coding the original text to visualize the machine-identified characters. Other features will need additional research, for example, suggest potentially problematic characters for the user to review.

## Additional files

Additional file 1: Original input. The original input used in the experiment. (TXT 296 kb)
Additional file 2: Normalized input. The normalized input used in the experiment. (TXT 308 kb)
Additional file 3: Gold standard matrix. The expert hand-curated matrix used as the ground truth to evaluate the ETC generated matrices. (XLSX 31 kb)
Additional file 4: Term categorization. This text file shows how the terms were categorized in the Review step of ETC Text Capture in the experiment. (TXT 12 kb)
Additional file 5: Matrix edits. List all the edits made in the ETC generated matrices. (DOCX 19 kb)

## Abbreviations

ASCII: American Standard Code for Information Interchange
CSV: Comma separated values
ETC: Explorer of Taxon Concepts
OTO: Ontology Term Organizer
PDF: Portable Document Format
XML: Extensible Markup Language
