# Supplementary material for: Introducing Explorer of Taxon Concepts with a case study on spider measurement matrix building
Source: BMC Bioinformatics. 2016 Nov 17;17:471. doi: 10.1186/s12859-016-1352-7 (PMC5114841; doi:10.1186/s12859-016-1352-7)
Supplement: Additional file 5: — Matrix edits. List all the edits made in the ETC generated matrices. (DOCX 19 kb) [file 12859_2016_1352_MOESM5_ESM.docx]

Experiment Procedure of Matrix Generation in Spider Case Study

All characters listed in the second column “characters affected” of both two tables were selected in Preview and Selection View of the Review step, which are the relevant body part characters with numerical measurements, named as pre-edit matrices. The third column “operations” recorded how we edited matrices and generated the post-edit matrices.

Table 4. Edit operations performed in the matrix generated from the original input.

| edit type | characters affected | operations | Edit Effort |
| --- | --- | --- | --- |
| Delete | quantity of leg [15], character of carapace [4], length of carapace [147], width of carapace [76], length of abdomen [133], length of sternum [168],  quantity of iii tibia/metatarsu [1], quantity of iii [8], quantity of leg-3 [1] | delete column | 9 |
|  |  |  |  |
|  |  |  |  |
|  |  |  |  |
|  |  |  |  |
|  |  |  |  |
| Rename | quantity of whole-organism [162] | Rename as "length of whole-organism (new)" | 1 |
|  | quantity of carapace (split, length) [139] | Rename as "length of carapace (new)" | 1 |
|  | quantity of carapace (split, width) [129] | Rename as "width of carapace (new)" | 1 |
|  | quantity of abdomen (split, length) [133] | Rename as "length of abdomen (new)" | 1 |
|  | quantity of sternum (split, length) [165] | Rename as "length of sternum (new)" | 1 |
|  | quantity of spiracle-epigastrium [138] | Rename as "distance of spiracle-epigastrium (new)" | 1 |
|  | quantity of spiracle-spinneret [155] | Rename as "distance of spiracle-spinneret (new)" | 1 |
|  | quantity of i tibia [189] | Rename as "length of leg i tibia (new)" | 1 |
|  | quantity of i metatarsus [189] | Rename as "length of leg i metatarsus (new)" | 1 |
|  | quantity of ii tibia [188] | Rename as "length of leg ii tibia (new)" | 1 |
|  | quantity of ii metatarsus [188] | Rename as "length of leg ii metatarsus (new)" | 1 |
|  | quantity of iii tibia [185] | Rename as "length of leg iii tibia (new)" | 1 |
|  | quantity of iii metatarsus [185] | Rename as "length of leg iii metatarsus (new)" | 1 |
|  | quantity of iv tibia [186] | Rename as "length of leg iv tibia (new)" | 1 |
|  | quantity of iv metatarsus [186] | Rename as "length of leg iv metatarsus (new)" | 1 |
| Merge | length of whole-organism (new) [162], quantity of body [1] | Merge into length of whole-organism (new)" | 1 |
|  | length of carapace(new) [129], quantity of prosoma(split, length) [37], quantity of thoracic-groove [6], quantity of cephalic-area [1], quantity of front [2], quantity of ocular-area(split, length) [2] | Merge into “length of carapace (new)” | 5 |
|  | width of carapace (new) [ 139], quantity of prosoma(split, width) [37], quantity of ocular-area(split, width) [1] | Merge into “width of carapace (new)” | 2 |
|  | length of palpal-tarsus [5] *, quantity of palpal-tarsus [57] | Merge into “length of palpal-tarsus” | 1 |
|  | length of abdomen (new) [133], quantity of opisthosomum(split, length) [44] | Merge into “length of abdomen (new)” | 1 |
|  | width of abdomen [2] *, quantity of opisthosomum(split, width) [7], quantity of abdomen (split, width) [128] | Merge into “width of abdomen” | 2 |
|  | quantity of sternum (split, width) [160], width of sternum [1] * | Merge into “width of sternum” | 1 |
|  | distance of spiracle-spinneret (new) [155], quantity of spiracle [1], quantity of spiracle spinneret [2] | Merge into “distance of spinneret-spiracle(new)” | 2 |
|  | quantity of epigastric-furrow [1], distance of spiracle-epigastrium (new) [138], quantity of epigastrium-epigastrium [1], quantity of epigastrium-spiracle [20] | Merge into “distance of epigastrium-spiracle(new)” | 3 |
|  | length of leg ii tibia (new) [188], quantity of ii (split, tibia) [5] | Merge into ”length of leg ii tibia(new)“ | 1 |
|  | length of leg ii metatarsus (new) [188], quantity of ii (split, metatarsus) [3] | Merge into “length of leg ii metatarsus(new)” | 1 |
|  | length of leg iv tibia (new) [186], quantity of iv (split, tibia) [4] | Merge into "length of leg iv tibia(new)" | 1 |
|  | length of leg iv metatarsus (new) [186], quantity of iv (split, metatarsus) [3] | Merge into "length of leg iv metatarsus(new)" | 1 |
| Total edits | | | 46 |

The numbers in “[]” indicate the number of values affected by an edit operation. Characters indicated with an “*” were retained without edits.

Table 5. Edit Operations Performed in the Matrix Generated from the Normalized Input.

| Edit type | characters affected | Operation | Edit Effort |
| --- | --- | --- | --- |
| Merge | length of whole-organism [161], length of body^$1^ [1] | Merge into *length of whole-organism* | 1 |
|  | length of carapace [147], size of carapace [4], length of prosoma^$2^ [37], length of ocular-area [1], length of thoracic-groove [2], length of cephalic-area [1] | Merge into *length of carapace* | 5 |
|  | width of carapace [152], width of prosoma^$3^ [37], width of ocular-area [1], width of thoracic-groove [2], width of cephalic-area [1] | Merge into *width of carapace* | 4 |
|  | length of abdomen [138], length of opisthosomum^$4^ [44] | Merge into *length of abdomen* | 1 |
|  | width of abdomen [128], width of opisthosomum^$5^ [6] | Merge into *width of abdomen* | 1 |
|  | location of spiracle [1], size of spiracle spinneret [2], distance of spinneret-spiracle[155] | Merge into *distance of spinneret-spiracle* | 2 |
|  | distance of epigastric-furrow [1], distance of epigastrium-epigastrium^$6^ [1], distance of epigastrium-spiracle [158] | Merge into *distance of epigastrium-spiracle* | 2 |
|  | length of leg-2 tibia [189], length of leg-2 [1],  size_or_shape of leg-2 (split, tibia)[2] | Merge into *length of leg-2 tibia* | 2 |
|  | length of leg-2 metatarsus [189], size_or_shape of leg-2 (split, metatarsus)[2] | Merge into *length of leg-2 metatarsus* | 1 |
|  | length of leg-iii tibia [186], length of leg-iii[1],  size_or_shape of leg-iii (split, tibia)[1] | Merge into *length of leg-iii tibia* | 2 |
|  | length of leg-iii metatarsus [186], size_or_shape of leg-iii (split, metatarsus) [1] | Merge into *length of leg-iii metatarsus* | 1 |
|  | length of leg-4 tibia [187], length of leg-4 [1],  size_or_shape of leg-4 (split) [2] | Merge into *length of leg-4 tibia* | 2 |
|  | length of leg-4 metatarsus [187], size_or_shape of leg-4 (split)[2] | Merge into *length of leg-4 metatarsus* | 1 |
| delete | length of leg [3] | delete *length of leg* | 1 |
|  | size of abdomen [3] (values are non-numerical, e.g. tiny) | delete *size of abdomen* | 1 |
|  | length of leg-iii tibia/metatarsu [1] | delete *length of leg-iii tibia/metatarsu* | 1 |
| Total edits | | | 28 |

The numbers in “[]” indicate the number of values affected by an edit operation. The 18 characters in the gold standard were all included in the machine-generated matrix. The characters superscripted with “$N” are considered equivalent to a corresponding character in the gold standard, either by their semantic equivalence (i.e.. $1), or by the experts’ decisions (i.e., $2-$6).
